# Classic Hodgkin Lymphoproliferative Diseases Clonally Unrelated to B-Chronic Lymphocytic Leukemia Successfully Treated with Bendamustine Plus Rituximab

**DOI:** 10.3390/cancers10090304

**Published:** 2018-09-03

**Authors:** Shinya Rai, Hirokazu Tanaka, Ko Fujimoto, Takahiro Kumode, Hiroaki Inoue, Yasuhiro Taniguchi, Yasuyoshi Morita, J. Luis Espinoza, Yoichi Tatsumi, Takashi Ashida, Ryota Matsuoka, Yukie Yara Kikuti, Naoya Nakamura, Itaru Matsumura

**Affiliations:** 1 Department of Hematology and Rheumatology, Kindai University Hospital, Faculty of Medicine, 377-2 Ohno-Higashi, Osaka-Sayama, Osaka 589-8511, Japan; htanaka@med.kindai.ac.jp (H.T.); sasfamily13@yahoo.co.jp (K.F.); kumode@med.kindai.ac.jp (T.K.); h.inoue@med.kindai.ac.jp (H.I.); m11049@med.kindai.ac.jp (Y.T.); moriyasu@med.kindai.ac.jp (Y.M.); luis@med.kindai.ac.jp (J.L.E.); anzen2@med.kindai.ac.jp (Y.T.); ashida@med.kindai.ac.jp (T.A.); imatsumura@med.kindai.ac.jp (I.M.); 2 Departments of Pathology, Tokai University School of Medicine, 143 Shimokasuya, Isehara, Kanagawa 259-1143, Japan; rmatsuoka.ub@gmail.com (R.M.); ki285273@tsc.u-tokai.ac.jp (Y.Y.K.); naoya@is.icc.u-tokai.ac.jp (N.N.); 3 Department of Pathology, Graduate School of Comprehensive Human Sciences, University of Tsukuba, 1-1-1 Tennodai, Tsukuba-shi, Ibaraki 305-8575, Japan

**Keywords:** lymphoproliferative diseases, Hodgkin lymphoma, chronic lymphocytic leukemia, clonality, Hodgkin/Reed-Sternberg cell, bendamustine

## Abstract

A 62-year-old male was diagnosed with chronic lymphocytic leukemia (CLL) and treated with a fludarabine-containing regimen which maintained the disease in a partial response. Nine years after diagnosis, a rapidly growing systemic lymphadenopathy was observed, and a biopsy specimen revealed the presence of typical Hodgkin/Reed-Sternberg (HRS) cells, surrounded by T-lymphocytes and CLL cells. Sequencing analysis of the germline complementary determining region 3 (CDR3) region of the *immunoglobulin heavy chain (IGH)* gene showed that the Hodgkin/Reed-Sternberg cells were clonally unrelated to the preexisting CLL cells and the HRS cells were composed of five different clones, leading to the molecular diagnosis of de novo lymphocyte-rich classic Hodgkin lymphoproliferative diseases (LPDs) with small lymphocytic lymphoma (SLL). As the initial treatment was neither effective for classic Hodgkin LPDs nor for SLL, Bendamustine, Rituximab (BR) was started and complete remission was achieved, which has continued for more than one year so far. BR may be a good therapeutic option for both entities without causing hematological toxicity.

## 1. Introduction

Lymphoproliferative diseases (LPDs) encompass a group of conditions characterized by the excessive proliferation of lymphocytes. The incidence of LPD is typically higher in individuals with immune dysfunction, such as those with HIV infection, those exposed to immunosuppressive agents or in the post-transplant setting [1]. Lymphoid proliferations in LPDs include a spectrum of pathologic changes ranging from reactive hyperplasia to malignant lymphoma (ML), including diffuse large B-cell lymphoma (DLBCL) and classic Hodgkin lymphoma (CHL). LPD cases fulfilling the histopathologic criteria for DLBCL have a polyclonal, oligoclonal, or monoclonal nature [2]. However, thus far there are no reports on the clonality of each Hodgkin/Reed-Sternberg (HRS) cell in classic Hodgkin LPDs. This is because of the technical limitations to examine the clonality of HRS cells from histologic sections at a single cell level.

Chemotherapy-related LPDs (CR-LPDs) are more frequently associated with chronic lymphocytic leukemia/small lymphocytic lymphoma (CLL/SLL) and approximately 10% of CLL/SLL cases develop aggressive ML (referred to as Richter syndrome) during their clinical course, with most cases representing DLBCL and less than 1% being CHL, also known as an CHL variant of Richter syndrome (CHL-RS) [3]. Richter syndrome can be divided into two groups: (1) transformation of CLL/SLL clone into aggressive ML referred to as “true” Richter transformation; (2) development of LPDs from a novel clone distinct from the initial CLL/SLL clone. Despite their different cell origin [3,4], CHL or classic Hodgkin LPDs arising in CLL/SLL cases share an aggressive clinical course and dismal prognosis [5,6], which is further complicated by the lack of defined treatment guidelines for these neoplasms.

We here report for the first time a case clinically diagnosed as CHL-RS that was clonally unrelated to a preexisting CLL/SLL clone. This case was molecularly diagnosed as classic Hodgkin LPDs with SLL, and the treatment with BR (Bendamustine, Rituximab) resulted in long-term complete remission of both entities without causing hematological toxicity.

## 2. Materials and Methods

Laser microdissection (Carl Zeiss MicroImaging, Jena, Germany) was utilized to perform the single cell isolation of HRS cells from hematoxylin stained of 4 um thick sections of formalin-fixed paraffin embedded tissue of a lymph node. The single HRS cells were micro-dissected and collected directly to *Taq* Extender buffer (Stratagene/Agilent, Santa Clara, CA, USA) and proteinase K (Roche, Mannheim, Germany) reaction mixture. The *immunoglobulin heavy chain* (*IGH*) region was amplified by semi-nested polymerase chain reaction (PCR) using Ampli Gold PCR Master Mix (Roche, Branchburg, NJ, USA), *Taq* Extender PCR Additive according to the manufacturer’s instructions and primers: 5′-AGGTGCAGCTGSWGSAGTCDGG-3′, as upstream consensus *V* region primer (*FR1C*); 5′-CTTACCTGAGGAGACGGTGACC-3′, as a consensus *J* region primer (*LJH*); 5′-GTGACCAGGGTNCCTTGGCCCC-3′, as a consensus *J* region primer (*VLJH*). The fidelity of the *Taq* DNA polymerase utilized was estimated to be 2.28 × 10^−5^ change/base/cycle (Thermo Fisher Scientific). After cleanup (USB ExoSAP-IT, Thermo Fisher Affymetrix, Waltham, MA, USA) PCR product was directly sequenced (ABI PRISM Model 3100, version 3.7, Applied Biosystems, Foster City, CA, USA).

## 3. Case Presentation

A 62-year-old male was diagnosed with CLL (Binet B) in 2006. He was initially treated with fludarabine alone, then with FCR (Flu, cyclophosphamide, Rituximab), and kept in partial remission. In February 2015, a rapidly growing systemic lymphadenopathy was observed, and laboratory findings revealed elevated levels of lactate dehydrogenase (LDH) (414IU/L) and C-reactive protein (5.40 mg/dL), as well as very high levels of soluble IL-2 receptor (8387 U/mL). The bone marrow (BM) aspirate showed a massive infiltration of small lymphocytes (>65% of total BM cells), and flow cytometry (FCM) analysis revealed that most of the small lymphocytes in the peripheral blood (PB) and BM were positive for CD5, CD19, CD20, CD23 and IG light chain, resembling the phenotypic feature of the original CLL cells. These small lymphocytes displayed a normal karyotype and had no deletions of p53 according to a fluorescence in situ hybridization (FISH) analysis. Reverse Transcription-Polymerase Chain Reaction (RT-PCR) analysis revealed 2300 copies of Epstein-Barr Virus (EBV) DNA per ml of whole blood at that time. In addition, the CLL cells were negative for Zeta-chain-associated protein kinase 70 (ZAP-70), a tyrosine kinase family normally expressed on T-cells and natural killer cells, whose expression on B-CLL served as a prognostic marker and an indicator for unmutated *IGH* variable regions of the CLL cells [4].

**Figure 1:**
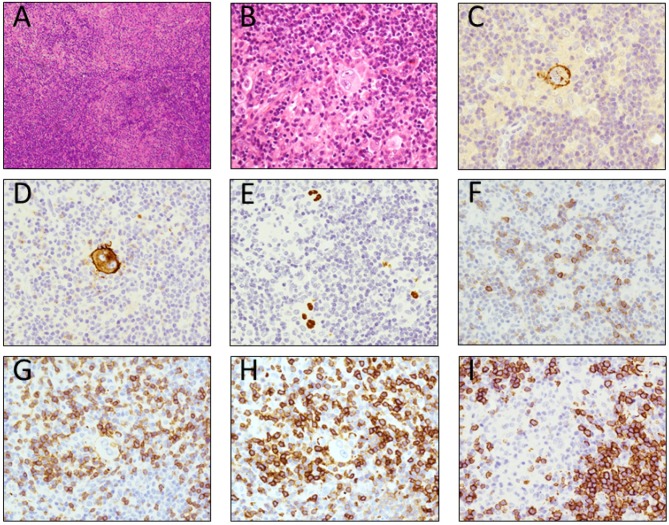
The pathologic examination of the axillary lymph node. (**A**) Hodgkin/Reed-Sternberg (HRS) cells with typical morphologic structure surrounded by small lymphocytes in the background of scattered small atypical lymphocytes: H-E staining, ×100 (A); H-E staining, ×400; (**B**) the HRS cells were positive for CD15 (**C**), CD30 (**D**), Epstein–Barr virus (EBER) (**E**). The small lymphocytes around the HRS cells were positive for CD2 (**F**), CD3 (**G**), CD5 (**H**). The background scattered atypical small lymphocytes were positive for CD5 (**H**), CD20 (**I**).

A fluorodeoxyglucose-positron emission tomography (FDG-PET) showed increased fluorodeoxyglucose (FDG) uptake in various lymph nodes, with a maximum standardized uptake value (SUVmax) higher than 8.5 in cervical, mediastinal and paraaortic lymph nodes. The right cervical lymph node was surgically excised, because SUVmax of the lesion was equivalent to that of the other lymph nodes and a histopathological analysis showed scattered Hodgkin/Reed-Sternberg (HRS) cells in the background of diffuse proliferation of small lymphocytes. Immunohistochemistry studies revealed that the HRS cells were positive for CD15, CD30 and epstein-barr encoded RNA (EBER). In contrast, the small lymphocytes around the HRS cells were positive for CD2, CD3 and CD5 but were negative for CD10, CD20 and CD23, consistent with the phenotype of normal T lymphocytes. In addition, CD5/CD20/CD23-positive small lymphocytes were intermingled, indicating B-CLL involvement (Figure 1). The patterns of rearranged *IGH* bands were the same amongst samples from the lymph node, the PB, and the BM by Southern blot (SB) analysis. 

**Figure 2:**
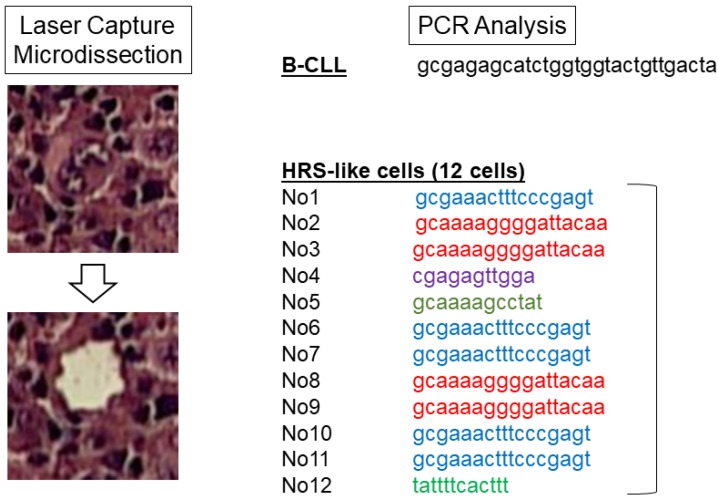
The sequencing pattern of the germline CDR3 region of the *IGH* gene. Left panel: representative laser microdissection of Hodgkin/Reed–Sternberg (HRS) cells within the lymph node is shown. Right panel: sequence trace in the germline complementary determining region (CDR)3 region of the *IGH* gene from a laser-captured B-CLL cell or each HRS cell is shown.

To elucidate whether HRS cells were clonally related to the original B-CLL clone, laser capture microdissection and semi-nested PCR studies of *immunoglobulin heavy chain (IGH)* were performed. Surprisingly, sequencing patterns of the germline CDR3 region of the *IGH* gene showed that the HRS cells were indeed clonally unrelated to those of B-CLL cells. Since the HRS cells were composed of five separate and distinct clones, a genetic diagnosis of de novo lymphocyte-rich classic Hodgkin lymphoma with small lymphocytic lymphoma (SLL) as made (Figure 2). However, because of rapid lymphoma progression with B symptoms, the patient was clinically diagnosed with CHL-RS.

Two courses of combination chemotherapy with doxorubicin, bleomycine, vinblastine, dacarbazine (ABVD) were unsuccessful, with a persistently high number of circulating B-CLL cells being documented in the PB, despite chemotherapy. In addition, the patient developed febrile neutropenia that required broad-spectrum antibiotics. Computed tomography studies indicated disease progression and therefore, salvage therapy with BR (90 mg/m^2^ Bendamustine on days one and two, 375 mg/m^2^ Rituximab on day one) was started. After six cycles of BR, complete remission was confirmed in both SLL and classic Hodgkin LPDs by FDG-PET, which has continued for more than one year so far.

## 4. Discussion

It has been thought that CHL is a B-cell neoplasm in nearly all instances, and that the HRS cells with rearranged immunoglobulin genes represent a clonal expansion from a single germinal center B cell [7]. In the case presented here, however, the sequencing patterns of the germline CDR3 region of *IGVH* gene from12 single HRS cells were clonally heterogeneous and were indeed unrelated to the preexisting CLL/SLL clone. These findings likely indicate an oligoclonal proliferation of the HRS cells, leading to the genetic diagnosis of classic Hodgkin LPDs with SLL.

Patients with hematologic malignancies treated with chemotherapy have an increased risk of developing LPDs [8,9], with CLL/SLL being the most frequently observed entity in these patients. Notably, patients treated with fludarabine appear to have a high predisposition for developing CR-LPDs with DLBCL accounting for the most common type of CR-LPDs arising in these cases, followed by polymorphic LPDs, and CHL [10]. Although CR-LPDs are not covered in the 2016 revision of the WHO classification [1], this entity harbors a spectrum of morphological features that are similar to those covered in the classification. Importantly, most of these LPDs fulfil the criteria for DLBCL and have a polyclonal, oligoclonal, or monoclonal nature [2]. However, there are no reports on the clonality of HRS cells in CR-LPDs, because of technical limitations inherent to single cell analysis. Several aspects of the pathogenesis of the CR-LPDs have not been clarified, however, current evidence suggest that several factors including host genetic susceptibility, an immunodeficient state, viral infection, (likely EBV), and chemotherapy-induced DNA damage may be causally implicated, and all these factors may not be mutually exclusive [11,12]. In this regard, CLL itself has intrinsic issues with immunity, and extrinsic chemotherapies may promote the overall degree of immunosuppression. In the patient described here, EBV was detectable not only in HRS cells but also in plasma at the time of diagnosis of the LPDs, which is consistent with previously reported cases of CR-LPDs and may therefore indicate that EBV likely plays a role in the development of classic Hodgkin LPDs [13].

Treatment guidelines for LPDs have not been established and most regimens utilized so far have proven to be ineffective. According to the largest study, the most common chemotherapy regimen for CHL-RS or classic Hodgkin LPDs was ABVD (Adriamycin, Bleomycin, Vinblastine, and Dacarbazine), and the median overall survival (OS) was only 8 months [5]. In addition, patients with CHL-RS who have a prior history of CLL and have been treated with fludarabine have a dismal prognosis compared to those who have not been exposed to fludarabine, regardless of the Ann Arbor stage or the histological type on transformation [6]. In the presented case, ABVD was ineffective and therefore, more aggressive approaches including stem cell transplantation were considered, however, these approaches were disregarded due to the physical frailty of the patient. Because cells from lymph nodes and the PB samples showed the same *IGH*-rearranged pattern according to the SB analysis, we speculated that the increasing lymphadenopathy was related to persistent PB B-CLL cells, however, we could not completely rule-out the possibility that some HRS cells remained to some extent in these lymph nodes. If the HRS cells were clonally related to the B-CLL/SLL cells, as hypothesized, those B-CLL/SLL cells could be specifically targeted by using novel agents such as Ofatumumab or Ibrutinib. Since Hodgkin LPDs and B-CLL/SLL were clonally unrelated, however, it was necessary to utilize a regimen capable of simultaneously targeting both entities.

Bendamustine is an alkylating agent with mild hematologic toxicity that has being used for the management of various B-cell malignancies including CLL. More recently it has been reported that Bendamustine has a clinical efficacy in refractory/relapsed CHL [14]. The largest report includes a series of 41 relapsed/refractory CHL patients treated with Bendamustine monotherapy. The overall response rate (ORR) was 58%, and the median OS was 21 months [15]. Furthermore, there were similar results (ORR 53%) observed in a phase two study for relapsed/refractory CHL patients that received Bendamustine monotherapy [16]. Although the achieved progression free survival (PFS) is modest, these studies strongly suggested that Bendamustine might be a good therapeutic option for refractory CHL, however, it must be noted that the BR combination may also promote CD4+ lymphopenia and associated immunosuppression. Therefore, potential EBV DNA reactivation should be carefully monitored in these patients.

## 5. Conclusions

In summary, we describe a first case of de novo lymphocyte-rich classical Hodgkin LPDs, clonally unrelated to a preexisting CLL clone with SLL, who achieved CR after six courses of BR with no hematologic toxicity, and this effect has continued for more than one year so far. Together, BR may be a good therapeutic option for both classic Hodgkin LPDs clonally unrelated to initial CLL clone and for SLL.
